# When the Eyes Go Dark: Transient Cortical Blindness After Primary Percutaneous Coronary Intervention

**DOI:** 10.7759/cureus.101507

**Published:** 2026-01-14

**Authors:** Soufiane Touiti, Oumayma El Khabote, Siham Hallab, Nada Fennich, Latifa Oukerraj, Mohamed Cherti

**Affiliations:** 1 Department of Clinical Cardiology, Cardiology B Hospital, Mohammed V University of Rabat, Rabat, MAR; 2 Department of Cardiac Catheterization, Cardiology B Hospital, Mohammed V University of Rabat, Rabat, MAR

**Keywords:** contrast-induced neurotoxicity, cortical blindness, diabetes mellitus, primary percutaneous coronary intervention, st-elevation myocardial infarction

## Abstract

Transient cortical blindness is a rare neurological complication associated with iodinated contrast media used during coronary angiography and percutaneous coronary intervention (PCI). Although uncommon, its sudden onset may mimic acute stroke, creating diagnostic uncertainty and significant anxiety. We report the case of a 49-year-old woman with a three-year history of type 2 diabetes mellitus who presented with prolonged retrosternal chest pain. ECG revealed ST-segment elevation in the inferior leads, consistent with inferoposterior ST-elevation myocardial infarction. Coronary angiography demonstrated an occlusion of the proximal right coronary artery, and successful primary PCI was performed with restoration of thrombolysis in myocardial infarction 3 flow. Near the end of the procedure, the patient developed acute bilateral blindness, preceded by visual fog, despite stable vital signs and the absence of focal neurological deficits. An urgent brain CT and subsequent MRI with angiographic sequences, performed four hours after symptom onset, were unremarkable. Supportive management with intravenous hydration was initiated, and the patient experienced complete visual recovery within 24 hours. The clinical presentation, normal neuroimaging, and rapid resolution were consistent with contrast-induced cortical blindness, a phenomenon attributed to transient blood-brain barrier disruption and direct neurotoxic effects of contrast agents on the occipital cortex. Although self-limited, recognizing this rare complication is crucial to distinguish it from ischemic stroke or posterior reversible encephalopathy syndrome and to prevent unnecessary interventions. Contrast-induced cortical blindness remains a reversible but impactful complication of PCI, and early recognition, exclusion of alternative diagnoses, supportive care, and patient reassurance are essential for optimal management.

## Introduction

ST-elevation myocardial infarction (STEMI) remains a major cardiovascular emergency requiring rapid reperfusion, ideally through primary percutaneous coronary intervention (PCI). While most complications of PCI are cardiac or vascular in nature, rare neurological complications can occur and may be particularly alarming. One such unusual event is transient cortical blindness related to iodinated contrast media [1,2].

Contrast-induced cortical blindness is characterized by sudden, bilateral loss of vision in the absence of ocular or focal neurological deficits, typically resolving spontaneously within hours to days [3]. The phenomenon is attributed to the neurotoxic effects of iodinated contrast agents on the occipital cortex, likely mediated by transient disruption of the blood-brain barrier. This complication is extremely rare, with only isolated case reports and small series described in the literature, which may contribute to its under-recognition in routine clinical practice. Early identification is essential, as its presentation can closely mimic acute ischemic stroke and may lead to unnecessary diagnostic or therapeutic interventions if not recognized. Although uncommon, its occurrence is increasingly reported in interventional cardiology and neuroradiology [4].

We report the case of a middle-aged diabetic woman who developed sudden bilateral blindness following successful PCI for inferior STEMI. The clinical course, diagnostic evaluation, and favorable outcome underscore the importance of recognizing this rare complication.

## Case presentation

**Figure 1:**
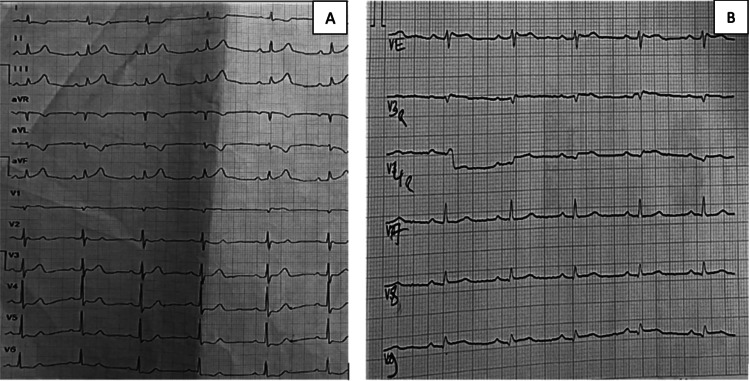
Admission 12-lead ECG and right precordial leads (A) Initial 12-lead ECG showing ST-segment elevation in the inferior leads (II, III, aVF) with reciprocal ST-segment depression in leads I and aVL. Mild ST-segment elevation is also noted in V5-V6, consistent with inferolateral extension. (B) Right precordial leads (V3R-V6R) demonstrate ST-segment elevation in V3R and V4R, confirming right ventricular involvement.

A 49-year-old woman with a three-year history of type 2 diabetes mellitus, treated with oral antidiabetic medications, presented to the emergency department seven hours after symptom onset with typical prolonged retrosternal constrictive chest pain. On admission, she was pain-free, and the clinical examination was unremarkable. ECG revealed ST-segment elevation in the inferior and posterior leads with reciprocal changes, consistent with inferoposterior STEMI (Figure 1).

**Figure 2:**
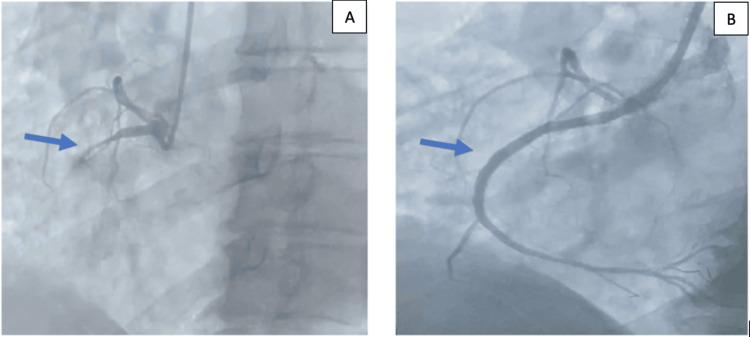
Initial angiography demonstrating complete occlusion of the proximal right coronary artery (A), followed by reperfusion with TIMI 3 flow after balloon angioplasty and stent implantation (B) (A) Initial coronary angiogram showing complete occlusion of the proximal RCA (arrow), consistent with the culprit lesion responsible for the inferoposterior myocardial infarction. (B) Post-angioplasty angiogram demonstrating successful revascularization with restoration of TIMI 3 flow throughout the RCA after stent implantation (arrow). RCA, right coronary artery; TIMI, thrombolysis in myocardial infarction

Emergency coronary angiography revealed an occlusion of the proximal right coronary artery (segment I). The patient underwent successful percutaneous transluminal coronary angioplasty with restoration of thrombolysis in myocardial infarction 3 flow (Figure 2).

**Figure 3:**
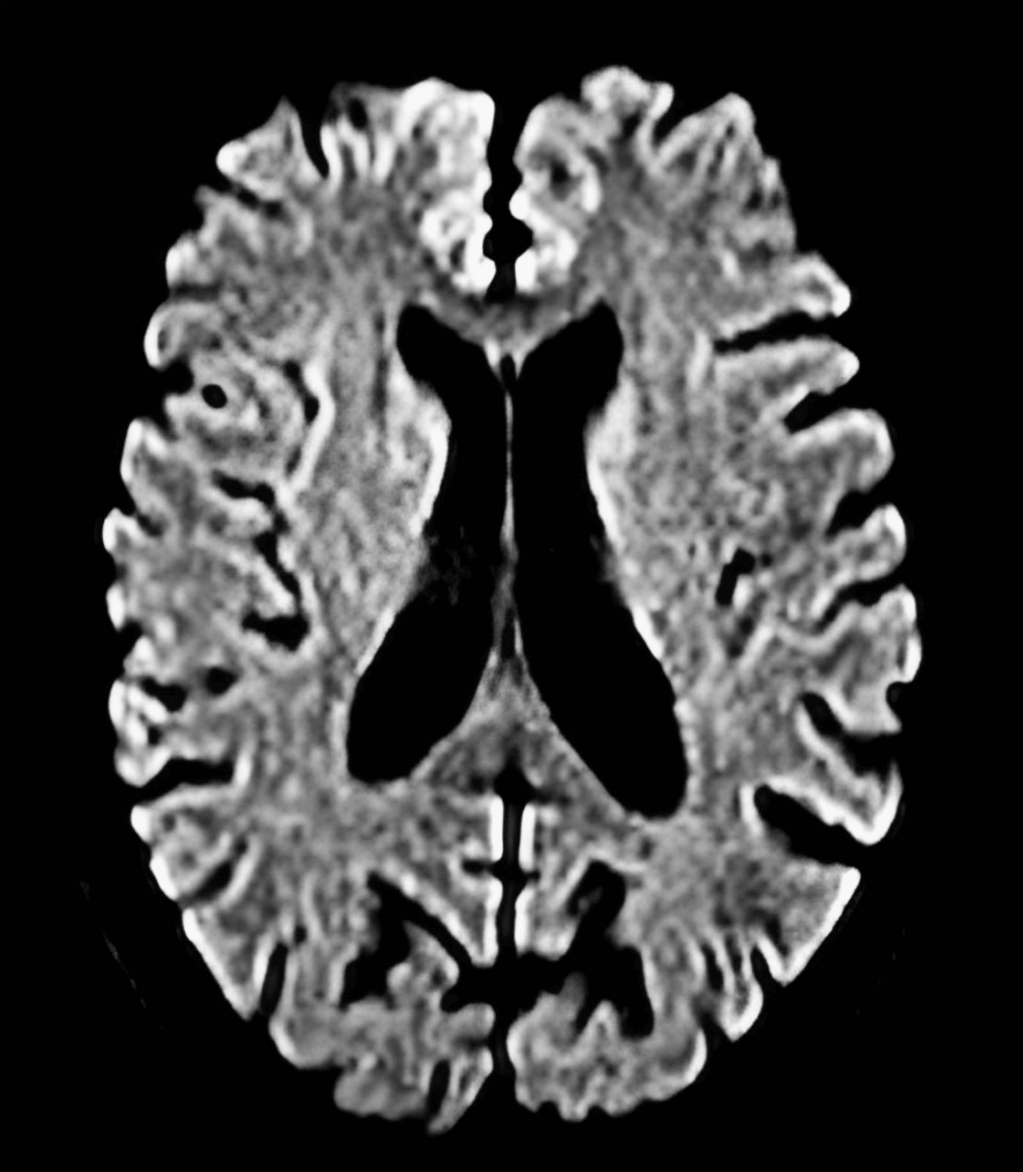
Brain MRI (DWI sequence) showing no evidence of acute ischemia Diffusion-weighted MRI performed four hours after the onset of visual loss demonstrates no restricted diffusion in the occipital lobes or elsewhere, excluding acute ischemic stroke. The normal appearance supports the diagnosis of transient contrast-induced cortical dysfunction rather than infarction.

Toward the end of the procedure, the patient experienced visual fog that rapidly progressed to sudden bilateral blindness. Hemodynamic parameters remained stable, with blood pressure at 131/67 mmHg, heart rate at 68 bpm, and capillary blood glucose at 130 mg/dL. Neurological examination revealed no focal motor or sensory deficits. An urgent brain CT scan was normal. Brain and orbital MRI with angiographic sequences, performed four hours after symptom onset, were also unremarkable (Figure 3).

Baseline laboratory tests demonstrated normal renal function, with a serum creatinine level of 8.5 mg/L and an estimated glomerular filtration rate of 71 mL/min/1.73 m². No electrolyte abnormalities were noted.

The patient was treated with intravenous hydration (750 mL of normal saline over 12 hours). Progressive visual improvement was observed, with complete recovery of vision within approximately 12 hours. The abrupt onset of bilateral blindness, normal neuroimaging, stable hemodynamic status, and rapid spontaneous recovery supported the diagnosis of contrast-induced transient cortical blindness.

## Discussion

Contrast-induced cortical blindness is a rare but recognized neurological complication of coronary angiography and PCI. It presents as acute bilateral vision loss without ocular or focal neurological deficits and typically resolves within hours to days. Although alarming, the prognosis is generally favorable if promptly identified [5].

The underlying mechanism is attributed to the transient disruption of the blood-brain barrier, allowing iodinated contrast to penetrate the occipital cortex and cause reversible cortical dysfunction. Diabetes, hypertension, renal impairment, advanced age, and large contrast doses are known risk factors. In our patient, diabetes may have predisposed them to microvascular fragility despite preserved renal function [6,7].

The differential diagnosis of sudden bilateral blindness after PCI is broad. Acute stroke must be excluded through urgent neuroimaging. Retinal artery embolism, while possible, would typically produce unilateral findings. Posterior reversible encephalopathy syndrome can mimic this condition but usually occurs with severe hypertension, seizures, and characteristic MRI lesions, which were absent in this case [8].

Management is primarily supportive, including hydration and close observation. Corticosteroids or osmotic therapy have been described, but evidence is limited. In most cases, as with ours, recovery is rapid and complete within 24-72 hours [9]. The clinical significance of this condition lies in its rarity and the potential for misdiagnosis as stroke, which could lead to unnecessary interventions. Awareness among interventional cardiologists is crucial, particularly in patients with risk factors. Preventive measures include minimizing contrast volume, ensuring adequate hydration, and selecting low- or iso-osmolar contrast agents when feasible [4].

This case reinforces that contrast-induced cortical blindness, although alarming, is typically benign and reversible. Early recognition, appropriate imaging, and patient reassurance are the cornerstones of management.

## Conclusions

Transient cortical blindness is an uncommon but dramatic complication of coronary angiography and PCI. Although its sudden onset may mimic severe neurological events such as stroke, its mechanism is usually related to reversible neurotoxicity of iodinated contrast media through transient blood-brain barrier disruption. In most cases, neuroimaging is normal, and the condition resolves spontaneously within 24 to 72 hours with supportive management.

This case highlights the importance of recognizing contrast-induced cortical blindness to avoid unnecessary diagnostic or therapeutic interventions, provide appropriate patient reassurance, and implement preventive strategies in individuals with predisposing risk factors. Raising awareness among interventional cardiologists and emergency physicians is essential to ensure prompt diagnosis and optimal patient care when confronted with this rare complication.
